# Autophagy Dysfunction and Oxidative Stress, Two Related Mechanisms Implicated in Retinitis Pigmentosa

**DOI:** 10.3389/fphys.2018.01008

**Published:** 2018-07-26

**Authors:** Mari-Luz Moreno, Salvador Mérida, Francisco Bosch-Morell, María Miranda, Vincent M. Villar

**Affiliations:** ^1^Department of Basic Sciences, Universidad Católica de Valencia San Vicente Mártir, Valencia, Spain; ^2^Departamento de Ciencias Biomédicas, Facultad de Ciencias de la Salud, Universidad Cardenal Herrera-CEU, CEU Universities, Valencia, Spain; ^3^Department of Medical Ophtalmology, Fundación para el Fomento de la Investigación Sanitaria y Biomédica de la Comunitat Valenciana, Valencia, Spain

**Keywords:** eye diseases, retinitis pigmentosa, oxidative stress, mitochondrion, autophagy

## Abstract

Retinitis pigmentosa (RP) is one of the most common clinical subtypes of retinal degeneration (RD), and it is a neurodegenerative disease that could cause complete blindness in humans because it ultimately affects the photoreceptors viability. RP afflicts an estimated 1.5 million patients worldwide. The retina is highly susceptible to oxidative stress which can impair mitochondrial function. Many retina pathologies, such as diabetic retinopathy and secondary cone photoreceptor death in RP, have been related directly or indirectly with mitochondrial dysfunction. The possible role of autophagy in retina and cell differentiation is described and also the implications of autophagy dysregulation in RP. The present review shows the crucial role of autophagy in maintaining the retina homeostasis and possible therapeutic approaches for the treatment of RP.

## Autophagy

Autophagy is taken from the Greek words auto “self” and phagy “eating” (self-eating) and was first described by Christian De Duve in 1963 (De Duve, 1963; Dodson et al., 2013) as a lysosome-mediated degradation process. Autophagy is a process that functions as an intracellular degradation system of cellular debris such as damaged proteins or organelles. Three major forms of autophagy have been described: CMA, microautophagy, and macroautophagy (Zhang et al., 2013).

**FIGURE 1:**
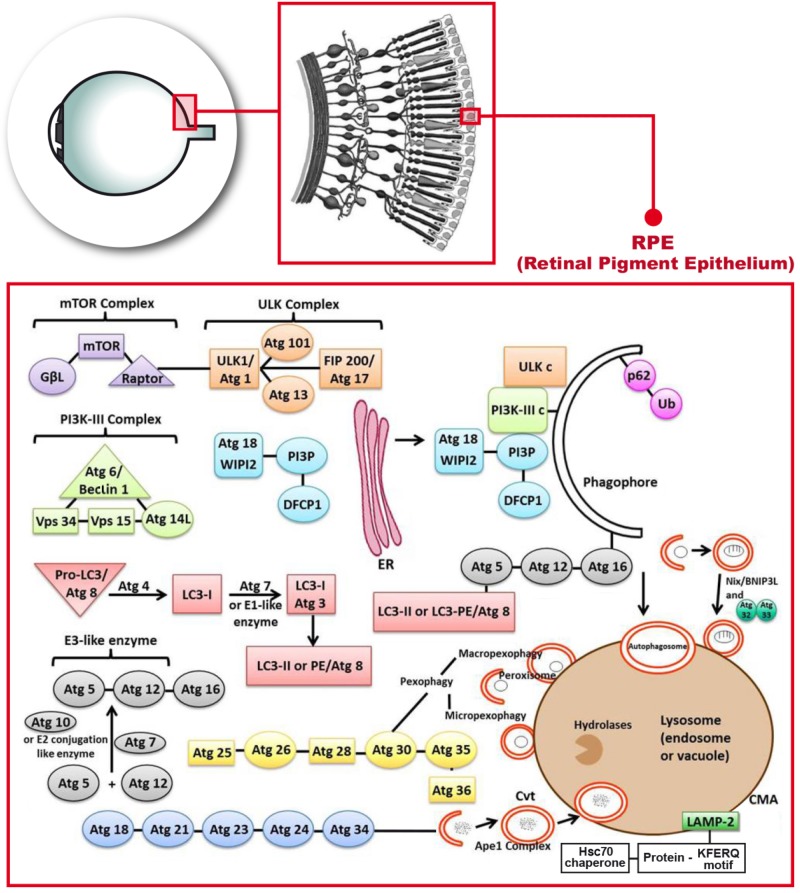
Molecular mechanisms describing the autophagy process that occur in the retinal pigment epithelium. Phosphatidylinositol-3-kinase-I (PI3K-I) is activated and this leads to the activation of the mechanistic target of Rapamycin (mTOR) triggering the dissociation of the serine/threonine-protein kinase complex (ULKc) that binds to the endoplasmic reticulum (ER). Subsequently, PI3K-III complex is activated and forms the phosphatidylinositol 3-phosphate (PI3P) pool in the rough ER. The Double FYVE-containing protein 1 (DFCP1) recognizes the new synthetized PI3P and forms the omegasome with the help of the ER. The WD repeat domain phosphoinositide-interacting protein 2 (WIPI-2) localizes to the omegasome-anchored phagophores. Then, Atg7 or E1 like (activation enzyme) together with Atg10 or E2 conjugation like enzyme activates the union of Atg5 and Atg12 (Atg5-Atg12) with Atg16. Atg7 also activates light chain 3-I (LC3-I) or Atg3, that forms LC3-II, also called Atg8 or LC3-phosphatidyl ethanolamine (LC3-PE). LC3-II gets inserted into the phagophore through the union to Atg5. Later on, P62 links ubiquitinated proteins or mitochondria to LC3 and the phagopore. Finally, the autophagosome formed, fuses with the lysosome to form the autolysosome.

Chaperone-mediated autophagy targets chaperones to proteins that contain the consensus sequence of the peptide Lys-Phe-Glu-Arg-Gln (KFERQ consensus sequence) (Dice, 1990; Bejarano and Cuervo, 2010). This sequence is recognized by a chaperone in the cytosol, the Hsc70 that targets the molecule to the surface of the lysosome. The complex binds to the lysosome-associated membrane protein type 2A (LAMP-2A), which in the CMA is like a receptor (Chiang et al., 1989; Cuervo and Dice, 1996) (**Figure 1**).

Microautophagy consists of the invagination of lysosomal membranes (Mijaljica et al., 2012; Martinez-Lopez et al., 2013). Microautophagy is involved in turnover of membrane proteins (Saksena and Emr, 2009). It takes place during nutrients recycling, as it happens also with macroautophagy. When it takes place after lipid degradation this process regulates membrane composition of the lysosomes and the glycogen delivery to lysosomes, and it also plays an important role in the cell under nitrogen restriction (Takikita et al., 2009; Li et al., 2012).

Macroautophagy is generally referred to as autophagy. It consists of the formation of double-membrane structures that contain damaged or partially degraded materials, including organelles such as mitochondria, and is implicated in bioenergetics regulation. The double-membrane structures are called autophagosomes. In the case of mitochondria, the unique mechanism that allows the complete degradation of them is known as mitophagy (Esteban-Martínez et al., 2017). Mitophagy was used by Lemasters for the first time in 2005 (Lemasters, 2005). Double-membraned autophagosomes enclose whole mitochondria (**Figure 1**), or selectively target the damaged areas. Autophagosomes then fuse with lysosomes for degradation. Furthermore, it is known that during starvation, mitochondria supply membranes for autophagosomes biogenesis (Hailey et al., 2010).

In autophagy, PI3K-I will get activated by the effect of ROS, lack of glucose or some type of exercise and it will subsequently lead to the activation of the mechanistic target of Rapamycin complex 2 (mTORc2) and in turn, it will activate the AKT/PKB (Sarbassov et al., 2004). At this point of the autophagy process, the AMPK activation will lead to the dissociation of the ULKc from the mammalian or mechanistic target of Rapamycin complex 1 (mTORc1), and ULKc finally binds to the ER (Biazik et al., 2015) (**Figure 1**).

The next step in autophagy implies the activation of PI3K-III complex and subsequently Atg14L recruits the complex to the ER. PI3K-III forms the PI3P pool in the rough ER (**Figure 1**). The Double FYVE-containing protein 1 (DFCP1) recognizes the new synthetized PI3P and forms the omegasome, which is an omega-like shaped scaffolding enriched with PI3P structures from the ER that serves as a platform for the biogenesis of autophagosome (Axe et al., 2008). The WIPI-2 localizes to omegasome-anchored phagophores, also called “isolation membrane”, which is a membrane cisterna that is involved in the first stages of the formation of the autophagosome (Polson et al., 2010) (**Figure 1**). This is still the initiation step. The conjugation cascade takes place at this point. The Atg7 or E1 like (activation enzyme) together with Atg10 or E2 conjugation like enzyme activates the union of Atg5 and Atg12 (Atg5-Atg12), which is the E3 like or ligation enzyme. Atg16 will get-together with these two Atgs (**Figure 1**). Atg16 has a scaffolding role in autophagy getting attached to the ER, and the rest of the steps are clearly indicated in **Figure 1**. Atg7 also activates light chain 3-I (LC3-I) or Atg3, that forms LC3-II, which is a MAP, or in eukaryotes it is Atg8 or LC3-PE. LC3 in mammals is an autophagosomal ortholog of Atg8 in yeast. LC3-I is transformed to a membrane-bound form which is LC3-II that corresponds in yeast to Atg8–PE. LC3-II is a lipidated form of LC3 and in mammals is used as an autophagosomal marker (Reggiori and Klionsky, 2013). LC3-II gets inserted into the phagophore through the union to Atg5 (Tanida et al., 2008). LC3-II is an ubiquitin like protein which is involved in the formation of autophagosomes.

The next step in autophagy is the elongation due to the conjugation cascade. P62 or SQSTM1 is an adaptor protein that links ubiquitinated proteins or even mitochondria to LC3. P62 gets attached to the phagophore (Joung et al., 1996). The sequestration of mitochondria is done through Nix or BNIP-3L (B-cell lymphoma-2 adenovirus E1B 19-kDa interacting protein 3-like) (**Figure 1**). This sequestration is required for selective clearance of mitochondria or for the actual mitophagy to occur. Nix/BNIP-3L is a mitochondrial protein. It can induce cell death through its BH3 domain and it can also induce autophagy through its LIR domain; mitophagy is mediated by Nix/BNIP-3L which acts as receptor (Zhang and Ney, 2009). The final step takes place with the fusion of the autophagosome with the lysosome to form the autolysosome that releases different molecules through permeases located in the membrane (Mizushima et al., 2002). The alternative path is the fusion of the autophagosome not with the lysosome but with the endosome to form the amphisome which is an intermediate vacuole.

## Oxidative Stress and Autophagy

ROS are generally small and highly reactive molecules that are formed naturally as a normal product of the oxygen metabolism in the mitochondrial electron-transport chain among other places (Scialo et al., 2017). The important role of mitochondria in the proper cell function which comprises ATP generation through aerobic respiration producing ROS as a byproduct is well known. ROS are a result of aerobic cellular metabolism during enzyme reactions that take place among other sites in the ER, in peroxisomes and in mitochondria (Waris and Ahsan, 2006). ROS can act as signaling molecules when they are at low physiological levels. However, some factors can modify these levels increasing the amount of ROS in the cell and generating oxidative stress (Indo et al., 2017).

Autophagy is activated during stress to protect organisms and so it has a protective role which is a survival response to oxidative stress, but also it can lead to some type of programmed cell death which is different from apoptosis (Mizushima et al., 2008). Impaired autophagy brings about a lot of oxidative stress and protein aggregation in many neurodegenative diseases (Chai et al., 2016). Thus, oxidative stress can either be a signal to activate autophagy or a way to inhibit it (Feng et al., 2005; Scherz-Shouval et al., 2007).

Oxidative stress is inseparably linked to mitochondrial dysfunction, as mitochondria are both generators of and targets for reactive species (Lee et al., 2012). Since the mitochondrial genome is devoid of histones it is not protected by them and it has a much higher mutation rate than the nuclear genome (Filosto et al., 2011). Oxidative stress can cause mutations in mitochondrial DNA, together with an imbalance in the mitochondrial respiratory chain, calcium homeostasis and excitotoxicicity, damages in membrane permeability and mitochondrial defense systems which are prominent causes of neuronal dysfunction and degeneration (Mattson, 2007; Guo et al., 2013). Maleki et al. (2013) studied the mitochondrial redox state as a quantitative marker of oxidative stress. (Maleki et al., 2013). Poor quality mitochondria may exacerbate the situation of oxidative stress generating a bioenergetics crisis (Murphy, 2009). Therefore, mitophagy is a necessary mechanism for mitochondria turnover that contributes to sustain cell survival and function. The process of mitophagy can downregulate the production of ROS in the mitochondria by removing the damaged ones which secrete excessive ROS (Tal et al., 2009; Wang et al., 2012).

## Retinitis Pigmentosa

The term RP describes a large group of hereditary retinopathies, very heterogeneous both from a genetic and from a clinical point of view. It is a rare disease and currently has no cure (Farrar et al., 2002).

RP mainly affects the rod photoreceptors which are responsible for night vision or for vision in dim light. However, once the rods have degenerated, the cones, responsible for the daytime vision and the main source of vision in human beings, also die, which leads to complete blindness (Phelan and Bok, 2000; Hartong et al., 2006). Although, the mutations that cause RP have been identified in different genes, the mechanisms that cause the death of photoreceptors are still unknown and currently no treatment is available (Kennan et al., 2005).

Finally, the blood circulation of the retina is reduced and the pigmentary epithelium degenerates. In late stages, the affected patients show an abnormal accumulation of pigment (clusters) in the peripheral retina. Symptoms typically begin in the early adolescence and severe visual dysfunction happens around 40–50 years of age (Sahni et al., 2011). However, in some cases there are patients with a progression of the disease of only about two decades. Conversely, other patients show a much slower disease progression that may never cause them blindness (Hamel, 2006).

The ideal treatment for RP would be to correct the genetic defect that causes the disease. In fact, gene therapy has been shown to be effective in genetically well-defined cases when it is applied in an adequate time frame (Maguire et al., 2008; Gorbatyuk et al., 2010).

Another therapeutic strategy is neuroprotection. The objective is to preserve the viability of the affected cells and to favor their survival mechanisms. With this type of treatment we can try to protect from death both the rods (carriers of the mutation in RP) and the cones, which degenerate in a secondary manner, independently of the mutation. Hence, it is very important to know the physiology and the possible alterations of the RP retina. Two mechanisms that have been lately related to the possible RP retinal changes are autophagy and different sources of oxidative stress.

### Autophagy in Retinitis Pigmentosa

Autophagy was first described in retina forty years ago (Remé and Young, 1977). Components of the pathway of autophagy have been found in almost every visual structure such as orbit, cornea, lens and retina. Moreover, important autophagy proteins have been detected in the different retinal layers and these include the INL and the outer one (ONL), the RPE and the GCL. These four retinal layers are characterized by their high metabolic consumption and they frequently suffer mitochondrial damage.

Autophagy alterations have been related to different eye diseases such as glaucoma, cataracts, age related macular degeneration (AMD), DR, ocular tumors, infections and thyroid-associated ophthalmopathy (Chai et al., 2016). Defective autophagy has also been related with RP, but the evidence regarding its role in the disease is controversial and/or there is not sufficient knowledge to do so. However, it is important to note that manipulation of autophagy may offer new therapeutic targets for all these visual pathologies.

Some theories have emerged that postulate autophagy may have beneficial effects in RP and other theories which suggest that autophagy may negatively influence photoreceptor death. This controversy may be explained by several factors: (1) RP is a very heterogenous disease with a lot of different mutations responsible for the disorder, and it is possible that the role or importance of retinal autophagy may be different depending on the mutation; (2) the heterogenity of the animal RP models and the fact that most of the results have been obtained in different animal models; (3) it is possible that autophagy may play different roles in death of rods and cones; finally (4) because it is possible that moderate levels of autophagy in the photoreceptors may be beneficial while excessive autophagy may be deleterious.

#### Theories That Support That Autophagy Is Decreased in RP

Photoreceptors are the light-sensing cells in the retina. Light is composed of photons that are small particles that represent units of energy. Visual transduction is the process by which a photon of light generates a nervous response in the photoreceptors. Visual pigments can be found in the apical portion of the external segments of the photoreceptors. The absorption of light causes a negative potential inside the photoreceptor cell. The initial event consists of the absorption of a photon by rhodopsin, which causes a conformational change of rhodopsin to its actived state. Rhodopsin, excited by light, catalyzes the transformation of a G protein called transducin. There is evidence that suggests that any alteration in the phototransduction pathways can lead to the death of photoreceptors. In this regard it has been postulated by Yao and coworkers that RP may be related to a significant accumulation of the visual transduction proteins (rhodopsin, transducin, etc.) because of a defective autophagic process. Their results demonstrate that visual transduction proteins are associated with autophagosome-specific proteins and that deletion of Atg5, which is an essential protein in the cascade of autophagy, induced a decrease in autophagy and an increase in rod cell death (Yao et al., 2016). These findings may be explained by the fact that autophagy may play a key role in photoreceptor homeostasis by degrading visual transduction proteins and preventing their accumulation.

Results from Zhou and collaborators also confirmed the importance of abnormal visual protein accumulation in RP. In their work, these researchers deleted the gene encoding the Atg5 protein only in rods, thereby decreasing autophagy, increasing the accumulation of transducin and inducing degeneration of rods (Zhou et al., 2015).

Kaushal (2006) suggested that rapamycin, which increases autophagy, may be useful in clearing abnormal proteins in RP (Kaushal, 2006). The results from Bhootada and coworkers are in agreement with these theories. They have confirmed that ATF4, an UPR transcription factor, governs different signaling pathways, such as oxidative stress and autophagy in various pathologies. ATF4 is capable of triggering both a pro-survival and cell death pathways and that is why there are some contradictions in the literature regarding its clinical application. ATF4 overexpression contributed to the photoreceptor cell loss, therefore the deletion of ATF4 in a rhodopsin mice model enhanced photoreceptor survival that was accompanied by an increase of autophagy (Bhootada et al., 2016) hence there is a therapeutic potential in controlling ATF4 expression in RP (Pitale et al., 2017).

One of the most widely used animal models of RP is the retinal degeneration 10 (rd10) mice. This mutant phenotype is inherited as an autosomal recessive gene. The mutation in the rd10 mice is located on chromosome 5 and is caused by a missense mutation in exon 13 of the PDE6β gene. The rd10 mouse model simulates the typical progression of an autosomal recessive RP in a better manner than other RP models since the loss of photoreceptor cells happens after the terminal differentiation of the retina. This is because the retinal degeneration in the rd10 mouse model is not caused by the absence of the PDE6β protein but by an insufficient expression and/or low activity of this enzyme unit. Rodriguez-Muela and collaborators in 2015 used this animal model to demonstrate that the expression of the autophagy marker LC3-II is reduced and autophagy flux is blocked in rd10 retinas. In addition, they demonstrated that LMP occurs before the peak of photoreceptor death in the rd10 retina. This suggests that normalization of this process could be a good therapeutic option (Rodriguez-Muela et al., 2015).

#### Theories That Suggest That Autophagy Increases Photoreceptor Cell Death in RP

Kunchithapautham and Rohrer (2007) found that genes responsible for apoptosis and autophagy are co-expressed in photoreceptors that suffer death and degeneration. They suggest that autophagy may initiate apoptosis and therefore, contribute to photoreceptor death (Kunchithapautham and Rohrer, 2007).

Bogéa et al. (2015) identified a transgenic Xenopus laevis model of RP. In this animal model, light-induced retinal degeneration caused by P23H rhodopsin occurs via cell death with autophagy (Bogéa et al., 2015). Chen and collaborators used a beclin 1± mouse and a rod Atg7 knockout mouse. The beclin 1± mouse developed intense photoreceptor degeneration under high light conditions. The decrease in Atg7 resulted in retinal degeneration under normal and high light. These authors concluded that under low levels of stress, autophagy is already activated and it may be beneficial but when stress levels are very high, they activate autophagy even more, which increases cell death and retinal degeneration (Chen et al., 2013). However, this is not that simple. Recently, Yang et al. (2017) showed that activation of SR1, a ligand regulated chaperone, protects cones in the rd10 mouse. Even though its mechanism is not completely understood, the authors showed how in the rd10 mice retina the SR1 knockout enhances at an early age the cytoprotective processes ER stress and autophagy, and this was done measuring the levels of their respective protein markers C/EBP homologous protein (CHOP) and LC3-II. But in later stages the S1R knockout in the rd10 mouse retina inhibits autophagy, activates Müller cell gliosis and necroptosis leading to cone and rod death (Yang et al., 2017). Wang et al. have recently suggested the activation of SR1 as a therapeutic pharmacological tool for this RP model (Wang et al., 2016).

#### Roles of Autophagy in the Death of Cones in RP

In RP death of rod photoreceptor cells is directly due to genetic mutations and death of cone photoreceptor cells is not related to these mutations but to death of rod photoreceptors. Some studies suggest that degeneration of rod photoreceptor cells is caused by autophaghy impairment (Rodriguez-Muela et al., 2015; Zhou et al., 2015), while others indicate that activation of autophagy seems to play a role in cone photoreceptor death (Punzo et al., 2009). The therapeutic consequences would be difficult to explain. Autophagy would have to be stimulated at the beginning of RP when there are still some rods and cones alive, and then when rods have died already autophagy would have to be inhibited to facilitate cone survival.

Autophagy may play a different role in rods and cones. In 2009 Punzo and coworkers suggested that degeneration of cones in RP occurs secondarily to the deficiency of nutrients. Their results demonstrate that many of the genes involved in cellular metabolism and especially those related to the mTOR signaling pathway were decreased in numerous experimental RP models. These reduced levels of mTOR would induce the activation of autophagy. The mTOR receptor inhibits autophagy. Tumor suppressor genes, such as Tsc1, trigger autophagy inhibiting the mTOR receptor. Therefore, the mTOR pathway may be the key one in regulating autophagy (Howell et al., 2013). The implications of Punzo’s results are particulatly promising because the death of cones in RP has long been a paradox, since most of the genes that cause RP are expressed selectively in rods.

Venkatesh and coworkers showed that activation of mTORc1 by loss of its negative regulator Tsc1 was sufficient to promote the survival of starving cones of rd1 mouse helping with glucose uptake. However, this caused an autophagy defect accumulating ubiquitinated proteins which was not due to inhibition of initiation but to accumulation of lysosomes, hence suggesting an end-stage defect leading to aminoacid shortage and so decreasing long term cone survival. Sporadically administered rapamycin, the mTORc1 inhibitor, was enough to correct the cone defects mediated by loss of Tsc1 because this negative regulator was not able to completely inhibit it and so autophagy was activated in a proper way in the absence of aminoacids. The PTEN is another negative regulator and its loss led also to the activation of mTORc1, but it did not affect autophagy and amino-acid metabolism, and so there was a long-term protection of cones as a consequence of this activation (Venkatesh et al., 2016).

As previously mentioned, the cones are responsible for the most important visual functions, so the knowledge of the mechanisms that trigger their degeneration is fundamental and could lead to the discovery of new and important therapeutic targets. Another advantage of the therapies, whose objective is to delay the death of the cones, is that they could be administered even in advanced stages of the disease, since it has been shown that even when 95% of the cones have been lost, the vision is still substantial (Punzo et al., 2009).

### Retina Vulnerability to ROS and Oxidative Stress in Retinitis Pigmentosa

Retina is one of the tissues more metabolically active. It contains a great number of mitochondria and it is under constant stress due to the photochemical reactions (Jarrett et al., 2008). As a consequence, retina is the highest oxygen (O_2_) consumption tissue of the organism and at the same time it is especially vulnerable to oxidative stress (Ames, 1992; Guo et al., 2014). Therefore, it is exposed to the possible oxidative damage from ROS (Winkler et al., 1999). Retinal oxygenation is unique in many ways due to the presence of two different sources of O_2_: the choroidal vascular bed and the retinal vasculature. Photoreceptors are exposed to a large gradient of O_2_ originated from the choroid toward the retina, high levels of polyunsaturated fatty acids and visible light exposure that can lead to a situation of oxidative stress (Hoang et al., 2002; Stone et al., 2008; Barbas-Bernardos et al., 2016; Carver and Yang, 2016).

Some ocular pathologies can exacerbate retina ROS damage as occurs in RP. It seems that rod cell death increases the oxidative stress in the retina enhancing the oxidative damage to cones in RP (Campochiaro and Mir, 2018). When rods die the oxygen consumption in the retina decreases and, because the blood circulation in the vessels of the choroid does not regulate itself, a situation of hyperoxia could cause oxidative damage (Bill and Sperber, 1990). This damage would affect the survival of retinal cells, including cones that undergo progressive oxidative damage that it would ultimately trigger their death by apoptosis and further exacerbate the death of rods (Usui et al., 2009). High levels of oxygen would favor the accumulation of ROS which would cause significant damage to lipids, proteins and DNA from different ocular structures (Usui et al., 2011; Punzo et al., 2012).

The literature is quite extensive on this area of the activation of autophagy which is associated with oxidative stress (Scherz-Shouval et al., 2007; Shacka et al., 2008). Furthermore autophagy has a potential cytoprotective effect in RCGs because its autophagy minimizes ROS levels and sustains mitochondrial function (Boya et al., 2016; Boya, 2017). One thing that both RGCs and photoreceptor cells have in common is that autophagy could precede apoptosis in the same cell. Autophagy stops being a cytoprotective mechanism when a critical point of stress is reached and so apoptosis gets activated and then by caspase cleavage autophagy gets inactivated (Frost et al., 2014).

These hypotheses led to the conclusion that treatment with antioxidants in different animal models of RP and clinical trials in humans had to be performed. It has been demonstrated in various clinical trials that vitamin A or diltiazem together with taurine and vitamin E improved visual loss in patients with RP or at least delayed the onset of the disease (Berson, 1993). Antioxidants may have beneficial effects for patients with RP, although the therapeutic success seems to be highly related to the genetic defect of the patient, the antioxidant used and the time when the antioxidant is administered. Studies in animals with antioxidants show results which are similar to those obtained with patients. In rd1 mice, it has been shown that the combination of antioxidants such as zeaxanthin, lutein, α-lipoic acid, GSH and Wolfberry extract was able to decrease the death rate of photoreceptors (Sanz et al., 2007). Carnosic acid, which is another potent antioxidant, has been recently shown as another agent to slow photoreceptor degeneration in rd10 mice (Kang et al., 2016). Progesterone is capable of delaying the death of photoreceptors in rd1 mice, acting through different protection mechanisms, such as increasing the concentration of the endogenous antioxidant GSH in the retina (Sánchez-Vallejo et al., 2015). In rd1 and rd10 mice treatment with α-tocopherol, manganese (III) 5,10,15,20-tetrakis(4-benzoic acid) porphyrin (MnTBAP), ascorbic and α-lipoic acid reduced the loss of both rods and cones (Komeima et al., 2007).

It can be concluded that despite the large number of studies on the use of antioxidants for the treatment of RP, the effects observed are highly variable. This could be due to the great variation that exists in the different experimental designs in terms of the antioxidant used, the time of administration, the dosage, the route of administration and so on. However, antioxidants have the important advantage that they can be taken orally as nutritional supplements and they are generally considered safe for use in adult patients, being able to improve vision at least partially or in a transient way.

## Conclusion

Autophagy is essential for the physiological functions of all the different structures of the eye. Lysosome dysfunction could be the cause of autophagy impairment eventually leading to eye disease. Even though there are no perfect treatments for many eye diseases, autophagy-regulating kinases like mTOR and AKT could be good candidates for the future treatment of these diseases.

All the studies presented in this review, partially help to highlight the importance of autophagy and oxidative stress as two related mechanisms involved in RP and the possible theories that may support these processes. In the present field of research, current data shows how autophagy and ROS signaling is involved during the beginning of RP and its progression, but future research will have to deal with the response to antioxidant therapy and other types of agents. The complex interaction between these two mechanisms have been shown but there are still some unanswered questions. It is known that according to the stage of RP the choice of antioxidants or any other treatment will be different because ROS or autophagy can activate or inhibit the applied therapeutic tool. Effective RP treatment may include antioxidant agents and/or autophagy modulators which might decrease the excess of ROS levels and regulate autophagy, respectively, but preserving the cytoprotective mechanism of ROS-induced autophagy. The therapeutic goal will be to regulate the balance between accumulation of ROS and ROS-induced autophagy or cell death.

The findings which are reviewed in this paper emphasize the need for obtaining new therapeutical approaches for RP. Understanding that nowadays it is not possible to cure this irreversible loss of vision due to the irreversible deterioration of rod photoreceptors and the RPE cells, different therapeutic targets will have to be applied in the future because of the genetic complexity of this eye disease. Only a multifactorial approach will be effective in finding adequate treatment.

## Author Contributions

M-LM, VV, and SM wrote the paper and conceived and designed the figures. FB-M and MM reviewed the paper.

## Conflict of Interest Statement

The authors declare that the research was conducted in the absence of any commercial or financial relationships that could be construed as a potential conflict of interest. The reviewer NK and handling Editor declared their shared affiliation.

## Abbreviations

AKT/PKB: protein kinase B
AMD: age-related macular degeneration
AMPK: AMP-activated protein kinase
ATF4: activating transcription factor 4
Atg5-Atg12: AuTophaGy proteins 5-12 complex
BNIP-3L: B-cell lymphoma-2 adenovirus E1B 19-kDa interacting protein 3-like
CHOP: CCAAT/enhancer binding protein (C/EBP) homologous protein
CMA: chaperone-mediated autophagy
DFCP1: Double FYVE-containing protein 1
DR: diabetic retinopathy
ER: endoplasmic reticulum
GCL: ganglion cell layer
GSH: glutathione
Hsc70: heat shock cognate protein of 70 kDa
INL: inner nuclear layer
LAMP-2A: lysosomal-associated membrane protein 2A
LC3: light chain 3
LC3-PE: LC3-Phosphatidyl ethanolamine
LIR: LC3-interacting region
LMP: lysosomal membrane permeabilization
MAP: microtubule associated protein
MnTBAP: Manganese (III) 5,10,15,20-tetrakis(4-benzoic acid) porphyrin
ONL: outer nuclear layer
PDE6β: phosphodiesterase 6β
PI3K-I: phosphatidylinositol-3-kinase-I
PI3K-III: phosphatidylinositol-3-kinase-III
PI3P: phosphatidylinositol 3-phosphate
PTEN: phosphatase and tensin homolog
RD: retinal degeneration
RGC: retinal ganglion cell
ROS: reactive oxygen species
RP: retinitis pigmentosa
RPE: retinal pigment epithelium
SQSTM1: Sequestosome-1
SR1: sigma-1 receptor
TOR/mTOR: mammalian target of rapamycin
Tsc1: tuberous sclerosis complex protein 1
ULKc: serine/threonine-protein kinase complex
UPR: unfolded protein response
WIPI-2: WD repeat domain phosphoinositide-interacting protein 2
